# Arboviruses as an unappreciated cause of non-malarial acute febrile illness in the Dschang Health District of western Cameroon

**DOI:** 10.1371/journal.pntd.0010790

**Published:** 2022-10-12

**Authors:** Innocent M. Ali, Valery P. K. Tchuenkam, Mia Colton, Victoria Stittleburg, Cedar Mitchell, Claudia Gaither, Kyaw Thwai, Daniel O. Espinoza, Yerun Zhu, Haaris Jamal, Autum Key, Jonathan J. Juliano, Tume B. Christopher, Anne Piantadosi, Jesse J. Waggoner, Matthew H. Collins

**Affiliations:** 1 Department of Biochemistry, Faculty of Science, University of Dschang, Dschang, West Region of Cameroon, Cameroon; 2 Rollins School of Public Health, Emory University, Atlanta, Georgia, United States of America; 3 Division of Infectious Diseases, Department of Medicine, Emory University School of Medicine, Atlanta, Georgia, United States of America; 4 Division of Infectious Diseases, University of North Carolina at Chapel Hill, Chapel Hill, North Carolina, United States of America; 5 Emory University, Atlanta, Georgia, United States of America; 6 Department of Pathology and Laboratory Medicine, Emory University School of Medicine, Atlanta, Georgia, United States of America; US Department of Homeland Security, UNITED STATES

## Abstract

Acute febrile illness is a common problem managed by clinicians and health systems globally, particularly in the Tropics. In many regions, malaria is a leading and potentially deadly cause of fever; however, myriad alternative etiologies exist. Identifying the cause of fever allows optimal management, but this depends on many factors including thorough knowledge of circulating infections. Arboviruses such as dengue (DENV) cause fever and may be underdiagnosed in sub-Saharan Africa where malaria is a major focus. We examined cases of fever in western Cameroon that tested negative for malaria and found 13.5% (13/96) were due to DENV, with 75% (9/12) of these being DENV serotype 2 infections. Two complete DENV2 genomes were obtained and clustered closely to recent isolates from Senegal and Burkina Faso. The seroprevalence of DENV in this region was 24.8% (96/387). Neutralizing antibodies to DENV2 were detected in all (15/15) seropositive samples tested. Chikungunya (CHIKV) is an arthritogenic alphavirus that is transmitted by *Aedes* mosquitoes, the same principal vector as DENV. The seroprevalence for CHIKV was 15.7% (67/427); however, CHIKV did not cause a single case of fever in the 96 subjects tested. Of note, being seropositive for one arbovirus was associated with being seropositive for the other (Χ^2^ = 16.8, p<0.001). Taken together, these data indicate that *Aedes*-transmitted arboviruses are endemic in western Cameroon and are likely a common but underappreciated cause of febrile illness. This work supports the need for additional study of arboviruses in sub-Saharan Africa and efforts to improve diagnostic capacity, surveillance systems, and arbovirus prevention strategies.

## Author summary

Acute illness with fever is common but can be challenging for clinicians to manage, particularly in resource-limited settings. In sub-Saharan Africa, malaria is a major cause of fever, but other causes of fever are poorly documented or monitored, which impairs optimal medical care to patients and implementation of public health interventions to control leading causes of disease. Viruses transmitted by mosquitoes are a prevalent and expanding problem throughout the tropics and beyond; however, there is concern that these infections frequently go undetected in sub-Saharan Africa. We discovered a previously unrecognized outbreak of dengue virus in western Cameroon by testing remnant samples from over 400 patients that presented with fever. Our results indicate that dengue has circulated in this region for decades with little recognition. This study adds important information about causes of fever in sub-Saharan Africa and advocates for increasing investment in surveillance systems and prevention strategies for mosquito-borne viruses.

## Introduction

Acute febrile illnesses (AFI) commonly prompt presentation to medical attention in sub-Saharan Africa and represent challenging clinical problems. There are numerous possible etiologies, and the availability of diagnostic testing for non-malarial causes is generally limited. [1–3] Malaria remains the predominant concern in the febrile patient, and empiric antimalarials are administered in many areas for undifferentiated fever. While missing a diagnosis of malaria could have grave consequences, overuse of antimalarials could promote resistance and confer unnecessary side effects, as well as delay optimal management of the true cause of the presenting illness. Studies of AFI in sub-Saharan Africa (SSA) have identified some patterns, but what is most notable is the heterogeneity of reported etiologies. [2] Thus, further development of public health capacity, including surveillance systems, is needed to inform the best decisions for clinical management and health resource allocation at the local level. To address gaps in epidemiologic knowledge in western Cameroon, we asked whether arbovirus (ARBV) infection was a common cause of non-malaria acute febrile illness (nmAFI).

ARBVs transmitted by the anthropophilic vector *Aedes aegypti* [4,5] include dengue (DENV), Zika (ZIKV) and chikungunya (CHIKV) viruses and represent a major global health problem. [6,7] DENV is estimated to cause upwards of 400 million infections each year, [8,9] with 3.6 billion people at risk of acquiring a DENV infection. [10,11] DENV was responsible for about 41,000 deaths in 2017, [12] with an economic cost estimated at 2.1 billion USD between 2000–2007 for the Americas. [13] However, the precise global distribution and burden of DENV remains highly uncertain. [14] In SSA, DENV is endemic in at least 34 countries. There is currently no report of routine DENV diagnosis and treatment in health systems in Africa despite evidence of continuous, ongoing DENV transmission in East and West Africa, [1,15,16] and more recently in central Africa. [17–19] Consequently, febrile children and adults are managed as presumptive cases of malaria or bacterial infections, which limits the attention and awareness for infections caused by DENV or other viruses. Indeed, over treatment of malaria in Africa is well documented. Up to 70% of patients in Cameroon [20] and 88% in Ghana who received an artemisinin combination were negative for malaria by rapid diagnostic test. [21] The entrenched clinical paradigm of managing undiagnosed febrile patients with antimalarials, the lack of guidelines for ARBV control programs, and the accumulating record of DENV transmission among travellers to and residents of Cameroon and other places in SSA [2,3,22–26] all point to the probability that DENV is an important public health problem in SSA. [27] In this study, we hypothesised that ARBV such as DENV represent an important but underappreciated etiology of febrile illness in SSA countries such as Cameroon and investigated the hospital prevalence of DENV infection in a cross-sectional acute febrile illness survey of patients suspected of having malaria infection in Dschang, West region of Cameroon.

## Methods

### Ethics statement

The study was reviewed and approved by the institutional review board (IRB) of the Cameroon Baptist Convention Health Board (FWA00002077), Protocol IRB2019-40. Written informed consent was administered in French or English based on participant preference, via an independent translator (who also spoke the local language Yemba) as needed. For children, informed consent was obtained from a parent or guardian.

### Study design and operations

#### Design

The analysis presented in this article was a secondary analysis of remnant samples collected during a parent study focused on malaria. The parent study was a prospective hospital based cross-sectional survey in the three main health facilities in the Dschang Health District in the West region of Cameroon: Dschang district hospital, St Vincent Catholic Hospital and Batsinglah Catholic Hospital.

#### Study population and site description

The parent study for this project was designed to study the species and genetics of parasites causing malaria in this region of Cameroon (S1 Text). In total, 431 patients were enrolled between June 12, 2020 –September 8, 2020. Selected demographic variables are reported in **Table 1**, and clinical symptoms are detailed in **S1 Table**. The Dschang health district with its 22 health areas has a surface area of about 1060 km^2^. It is a tropical, semi-urban environment at an elevation of 1380-1400m above sea level. The rainy season lasts mid-March–mid October.

**Table 1:** Sociodemographic characteristics of study population and association with DENV infection outcomes.

	RT-PCR+ DENV (N = 96)			DENV IgG+ (N = 387)		
	*Positive*	*Negative*	X^2^	*Seropositive*	*Seronegative*	X^2^
Number of participants who tested positive n(%)	13 (13.54)	83 (86.46)		96 (24.81)	291 (75.19)	
Demographics						
***Age***						
<10	2 (18.18)	9 (81.82)	0.8806*	7 (14.58)	41 (85.42)	0.0687
10–14	0 (0)	6 (100)		3 (17.65)	14 (82.35)	
15–49	8 (13.56)	51 (86.44)		60 (24.29)	187 (75.71)	
>50	3 (15)	17 (85)		26 (34.67)	49 (65.33)	
***Gender***						
Female	7 (11.67)	53 (88.33)	0.5453	56 (24.78)	170 (75.12)	0.9882
Male	6 (16.67)	30 (83.33)		40 (24.84)	121 (75.16)	
***Level of Education***						
Primary	6 (12.77)	41 (87.23)	0.9235*	56 (30.27)	129 (69.73)	0.0443
Secondary	2 (12.5)	14 (87.5)		7 (15.56)	38 (84.44)	
Tertiary	5 (15.15)	28 (84.85)		33 (21.02)	124 (78.98)	
***Presence of Water Source (Within 2 min*. *walk)***						
Yes	1 (5.56)	17 (94.44)	0.4505*	15 (29.41)	36 (70.59)	0.3846
No	12 (15.79)	64 (84.21)		78 (23.78)	250 (76.22)	
Don’t know	0 (0)	2 (100)		3 (37.50)	5 (62.50)	
***Type of Water Source***						
Stream	0 (0)	4 (100)	--	5 (41.67)	7 (58.33)	--
Pond/Lake	0 (0)	7 (100)		3 (17.65)	14 (82.35)	
Swamp/Marsh	1 (14.29)	6 (85.71)		7 (35)	13 (65)	
No Answer	12 (15.79)	64 (84.21)		78 (23.64)	252 (76.36)	
Don’t know	0 (0)	2 (100)		3 (27.27)	5 (72.73)	

*Fisher’s exact test was used

#### Recruitment of study participants and biospecimens

Inclusion criteria of the parent study included fever (axillary temperature ≥37.5°C or self-reported history of fever) in past 24 hours without signs and symptoms suggestive of severe malaria. Participants were excluded if they had used anti-malarial medicine in the past 14 days. Dried blood spots (DBS) were collected via finger prick. For serologic studies, plasma proteins were eluted from DBS as previously described. [23,28,29] DBS eluate was heat inactivated for 30 minutes at 56°C and stored at 4°C for up to 1 week or at -20°C until use. One 6-mm hole punch of each DBS was reserved for molecular testing.

#### Laboratory assays and cartography (see S1 Text for greater detail)

Samples were tested as extensively as allowed by specimen quantity and assay costs. Testing of 96 malaria-negative fever cases by real-time reverse transcriptase-polymerase chain reaction (rRT-PCR) was prioritized, followed by serologic testing of all samples with remaining material for DENV and CHIKV. Genomic sequencing could be accomplished from the same nucleic acid extraction as rRT-PCR testing.

#### rRT-PCR testing

Total nucleic acids were extracted from DBS on an EMAG instrument (bioMérieux, Durham, NC) by first placing 6mm punches into 400μL of Nuclisense lysis buffer and incubating overnight at room temperature on a tube rocker. Eluted nucleic acid (5μL) was immediately tested by multiplex rRT-PCR for ZIKV, CHIKV, and DENV, [30] and DENV-positive samples were subsequently tested in a DENV multiplex assay to determine the serotype (**S1 Fig**). [31,32]

#### DENV genome sequencing and phylogenetic analysis

Seven samples underwent unbiased metagenomic sequencing followed by DENV-specific analysis. Reads underwent metagenomic classification and DENV genome assembly. For phylogenetic analysis, all complete DENV2 genomes were downloaded from NCBI on 7/3/22 (N = 3,229), and a maximum likelihood phylogenetic tree was constructed as described in S1 Text. After Cameroon sequences were confirmed to cluster with other DENV2 sequences of the Cosmopolitan genotype from West Africa (**S2 Fig**), a subset of 29 West African DENV2 Cosmopolitan sequences were used for further analysis (**S2 Table**). Sequence GQ398264 (Indonesia, 1976) was included as an outgroup. Maximum-likelihood and time-scaled Bayesian phylogenetic analyses were performed as described in the Supplementary Methods.

#### Viruses and cells

DENV WHO reference strains DENV1-4 and CHIKV strain 181/clone25, [33] which is a live attenuated vaccine strain compatible with BSL-2 work, were used. DENV stocks were prepared in C6/36 *Aedes albopictus* mosquito cells (American Type Culture Collection (ATCC) no. CRL-1660) and CHIKV stocks in Vero *Cercopithecus aethiops* monkey cells (ATCC no. CCL-81).

#### Antigen capture IgG ELISA

Binding IgG to DENV or CHIKV was measured by antigen capture ELISA as previously described. [34,35] Virus-reactive monoclonal antibody was used to capture virus antigen. Serum IgG binding to viral antigen was detected by an alkaline phosphatase-conjugated goat anti-human IgG secondary Ab and *p*-nitrophenyl phosphate substrate. Absorbance at 405 nm (optical density, OD) was measured. The cut off for positivity was calculated for each plate as the average OD of negative control serum + three standard deviations + 0.1. [23,28,29]

#### Neutralization assays

Neutralization titers were determined by 96-well micro focus reduction neutralization test (microFRNT) as previously described. [23,36,37] Serial dilutions of DBS eluate were mixed with approximately 75–100 focus-forming units of virus. After 24–72 hr (depending on virus), intracellular viral protein was detected by 4G2 [38] (for DENV2) or CHK-48 [39] (for CHIKV), foci were quantitated, and neutralizing antibody titers calculated (GraphPad Prism 7).

#### Map creation

Data were summarized by study site (**S3 Table**) and were mapped to study site locations using pie charts. All maps were made using ArcGIS version 10.7.1 with Cameroon boundary shapefiles obtained from GADM.org.

### Data management and statistical analysis

Clinical and sociodemographic data were collected at enrolment and were entered into the study database. Descriptive statistics were used to report prevalence of DENV cases and DENV and CHIKV seroprevalence. Associations between DENV infection outcomes and sociodemographic data were investigated by analysing cleaned data in Statistical Analysis Software (SAS). A logistic regression was performed to analyse the relationship between the parameters of interest and each of the infection outcomes. However, due to the highly dimensional data, the analysis model was prone to overfitting. Thus, a Chi-square test was conducted to compare the categorical variables to the outcome of interest. Fisher’s exact test was conducted for variables with less than 5 observations when appropriate.

## Results

### ARBV infection

Of 96 subjects with fever and negative malaria testing, 13 (13.5%) tested positive for DENV by ZCD assay [30] (**Table 2**). DENV2 was identified in 9 samples (75%), and DENV3 was identified in 4, with a single sample testing positive for both serotypes. No samples were positive for ZIKV or CHIKV RNA, and all 96 samples yielded a positive signal for the RNase P target (**S4 Table**). One sample could not be serotyped.

**Table 2:** rRT-PCR results for DBS samples positive for DENV RNA.

	ZCD Assay				DENV Multiplex Assay				
Sample Number	DENV	CHIKV	ZIKV	RNase P	DENV-1	DENV-2	DENV-3	DENV-4^a^	Final Result
DSG2020_061	19.67	N	N	22.91	N	21.92	N	N	**DENV-2** ^b^
DSG2020_345	22.34	N	N	24.61	N	24.95	N	28.40	**DENV-2** ^b^
DSG2020_346	35.58	N	N	26.93	N	38.74	N	41.10	**DENV-2**
DSG2020_007	37.03	N	N	24.30	N	39.24	N	40.52	**DENV-2**
DSG2020_262	37.55	N	N	24.15	N	38.15	N	41.11	**DENV-2**
DSG2020_013	37.95	N	N	24.24	N	38.46	N	41.66	**DENV-2**
DSG2020_012	38.68	N	N	24.22	N	40.80	N	41.17	**DENV-2**
DSG2020_341	40.59	N	N	23.62	N	43.09	N	42.69	**DENV-2**
DSG2020_003	35.03	N	N	23.51	N	36.51	40.13	39.54	**DENV-2/-3**
DSG2020_429	34.95	N	N	27.87	N	N	37.23	N	**DENV-3**
DSG2020_263	39.96	N	N	24.98	N	N	36.88	N	**DENV-3**
DSG2020_197	42.90	N	N	25.96	N	N	38.43	N	**DENV-3**
DSG2020_075	41.56	N	N	26.45	N	N	N	N	**Negative**

Abbreviations: CHIKV, chikungunya virus; DENV, dengue virus; ZIKV, Zika virus

^a^Known cross-reactions occur with DENV-2, results in a low-fluorescence curve that crosses the threshold in DENV-4 channel.

^b^Whole genome sequences obtained

**Fig 1:**
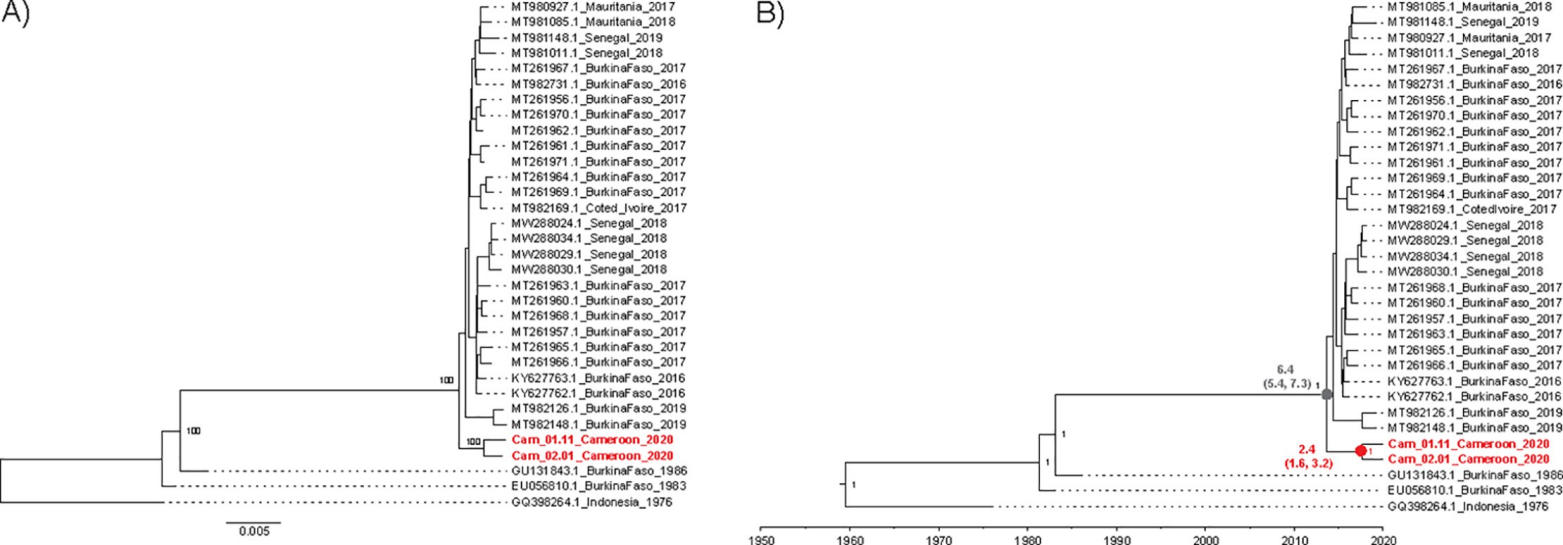
Phylogenetic analysis of DENV2 Cosmopolitan genotype sequences from West Africa. A) Maximum likelihood tree. Scale bar indicates genetic distance. Nodes in the ancestral lineage of Cameroon sequences are labeled with percent bootstrap support. B) Time-scaled maximum clade credibility tree. Nodes are labeled with posterior probability for the most recent common ancestor of Cameroon sequences (red) and its most proximal ancestor with other West African sequences (gray). Text boxes indicate the median node age and 95% HPD in years prior to 2020.

Samples with the lowest Ct values for DENV2 were sequenced. Approximately 1 million unbiased metagenomic sequencing reads were generated per sample (range 850,949 to 1,097,048, **S5 Table**). Complete DENV genomes were assembled from samples DSG2020_345 and DSG2020_061, confirming serotype 2. No DENV reads were detected in the other five samples (**S5 Table**), which can be attributed to their relatively low DENV RNA content (C_T_ values ranging from 34.95 to 42.90 in the ZCD assay), combined with our low-depth, unbiased sequencing approach; although a DENV-targeted approach likely would have yielded some DENV reads, it would be unlikely to allow full-genome sequencing and phylogenetic analysis for low-input samples such as these. Although one sample (DSG2020_346) contained GB virus C, no other potential pathogens were identified by metagenomic analysis. The two DENV2 genomes belonged to the Cosmopolitan genotype, and fell within a sub-lineage with sequences that have been circulating in West Africa for 20–35 years. [40,41] Interestingly, these sequences were distinct from and ancestral to sequences obtained from Burkina Faso, Cote d’Ivoire, and Senegal between 2016 and 2019 (**Fig 1A**). The Cameroon sequences, which were from samples collected in 2020, shared a common ancestor in approximately 2017 and diverged from other West African sequences in approximately 2013 (**Fig 1B**).

**Fig 2:**
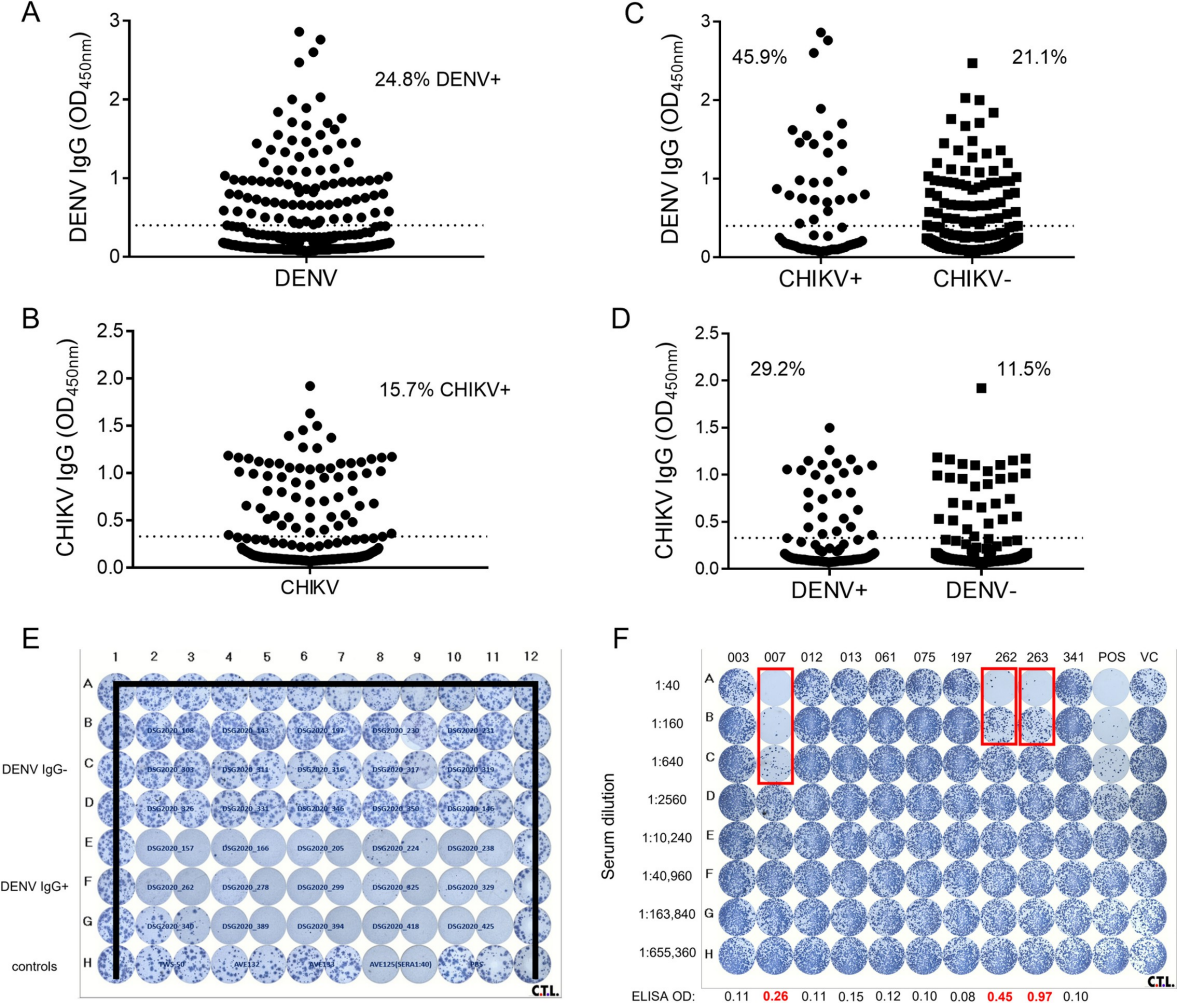
Seroprevalence (% shown on graph) for DENV (A, n = 387) and CHIKV (B, n = 427) by IgG ELISA are shown. The assay cutoff was determined by negative controls on each plate; a single dotted line, which is the average OD cutoff for all plates, is shown for better visualization. C) DENV IgG positivity is shown for samples (n = 383) grouped by CHIKV serostatus, and D) CHIKV IgG positivity is similarly shown for samples (n = 383) grouped by DENV serostatus. E-F) Neutralizing antibodies were assessed for DENV (E) and CHIKV (F) by focus reduction neutralization testing (FRNT). E) A subset of sera was selected based on DENV IgG serostatus (n = 15 DENV IgG- in upper portion of graph and n = 15 DENV IgG+ in lower portion of graph) and tested at a single dilution (1:80) in duplicate for neutralization of DENV2. AVE123 is a positive control (run at 1:40) and negative controls included TWS50, AVE132, and AVE133. All other wells forming the perimeter of the plate received virus only (marked through with thick bar). F) A subset of sera (n = 10) was selected based on CHIKV IgG serostatus (n = 7 CHIKV IgG- and n = 3 CHIKV IgG+) and tested over eight 4-fold dilutions in singleton for neutralization of CHIKV. CHIKV IgG ELISA results (OD, optical density) is shown across the bottom of each column, Positive ODs (OD>0.2) are shown in red.

To better understand the extent of ARBV transmission in the region, we tested for IgG antibodies to DENV and CHIKV, which revealed a seroprevalence of 24.8% (96/387) for DENV and 15.7% (67/427) for CHIKV (**Fig 2A–2D**). The ELISA testing approach was validated by testing a small random subset of samples for neutralizing antibodies (**Fig 2E and 2F**). All 15 DENV IgG+ samples exhibited neutralizing antibodies to DENV2, the most prevalent DENV serotype identified among non-malarial acute fever cases tested by rRT-PCR. To assess for coherence in ARBV infection, we analyzed the proportion of samples positive for one virus grouping by serostatus for the other virus (**Fig 2C and 2D**). There was a significant relationship between DENV and CHIKV seropositivity (χ^2^ (1, n = 383) = 16.777, p≤0.00005), consistent with the hypothesis that risk for multiple *Aedes*-borne viruses would be concentrated among some individuals.

### Risk factors for ARBV infection

**Fig 3:**
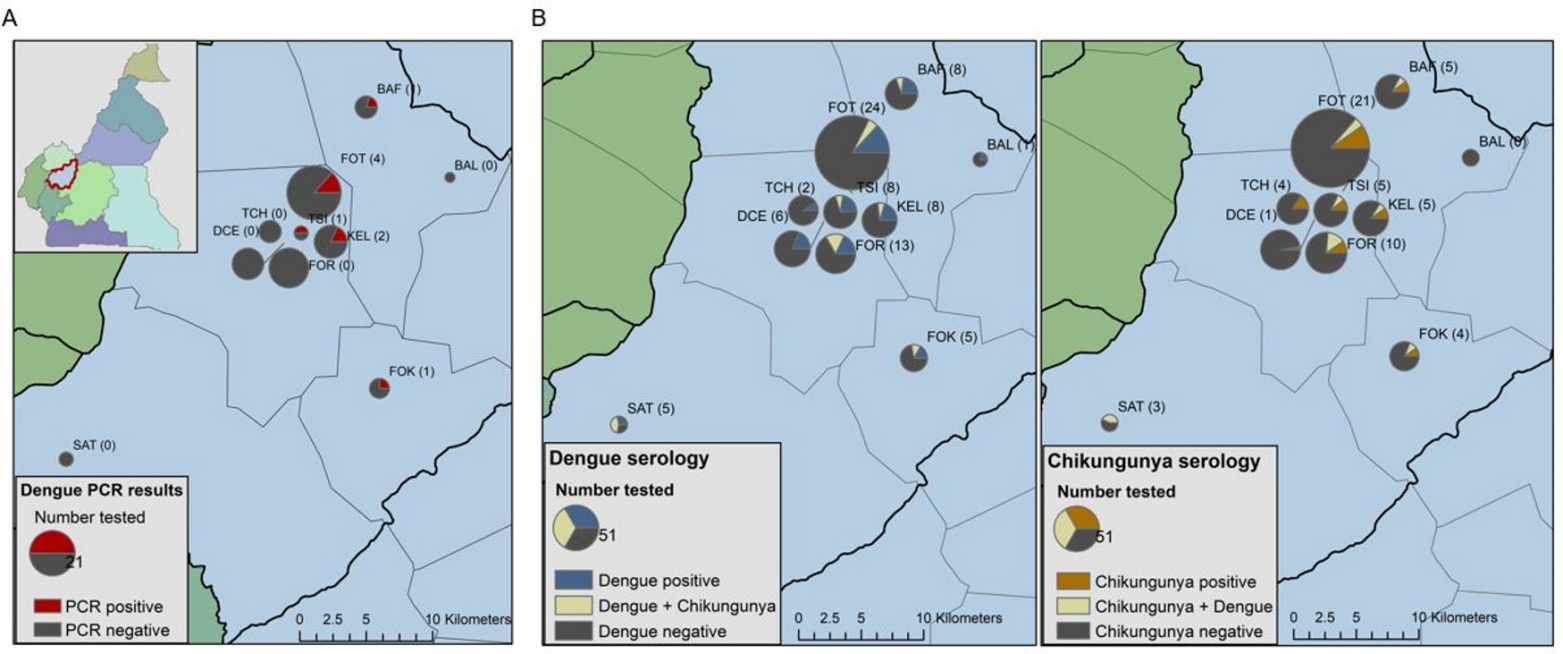
Distribution of rRT-PCR-positive DENV cases (A), and DENV (B), and CHIKV (C) seroprevalence were mapped by study site in Dschang Health District. Pie chart sizes were proportional of the number of samples tested for each assay. The abbreviated name of each study site appears next to each point with the number of samples positive for DENV via rRT-PCR (A), DENV IgG (B) or CHIKV IgG (C) in parentheses. For the DENV rRT-PCR results, sections of the chart corresponded to positive versus negative rRT-PCR results; for the DENV and CHIKV serology charts, sections corresponded to single infections (DENV or CHIKV only), DENV and CHIKV co-infections, and no DENV or CHIKV detected.

There was no clear geographic clustering by site for DENV cases or seroprevalence (**Fig 3A and 3B**). A Chi-square independence test showed that there were no significant differences between the sociodemographic factors and the epidemiologic outcomes of DENV case or seropositivity (p> 0.05) (**Table 1**). Analysis of a larger sample may reveal interesting differences in seroprevalence stratified by age, but no statistically significant relationships could be detected in the sample available for this study for age and DENV seroprevalence, χ^2^ (3, N = 387) = 7.10, p = 0.0687.

## Discussion

In this study, we demonstrated that DENV is clearly an important cause of AFI in SSA (at least in Cameroon). However, there may be substantial local and regional heterogeneity. Our study captured an outbreak driven primarily by DENV2 during 2020. The age distribution of DENV seroprevalence is consistent with endemic or intermittent transmission of DENV for at least several decades in the region. The seroprevalence of CHIKV further indicates that local ecology is amenable to human infection by multiple viruses transmitted by *Aedes aegypti*. Our two DENV2 genomes are important contributions to the scant phylogeographic data on DENV in SSA and suggest that a distinct lineage of the Cosmopolitan genotype of DENV2 may have been circulating unrecognized in western Cameroon for several years.

Our study adds to the limited existing evidence of DENV (including serotypes 1, 2 and 3) transmission in Cameroon in several regions. [3,17,42–51] CHIKV has been less commonly reported in Cameroon, [48,52–54] with exception of a notable outbreak in 2006. [53,54] The modest seroprevalence (~15%) of CHIKV but lack of cases in the AFI cohort illustrates a few important points. It is characteristic of many ARBV to exhibit unpredictable periodic outbreaks with intervening periods of low or absent transmission. The absence of detection of an ARBV like CHIKV in a single cohort study does not exclude this virus from being an important consideration in the etiology of AFI in this region. In fact, the seroprevalence data from our study and the sustained circulation of CHIKV in certain regions of Africa indicate the relevance of this pathogen. The second point is that diagnostic capacity is essential to support clinical practice and public health management of the myriad causes of fever in SSA. The association of DENV and CHIKV seropositivity strongly argues that common risk factors exist for *Aedes*-borne ARBV in this area, and there are several reasons why a comprehensive understanding of ARBV transmission dynamics is critical. As demonstrated by our study, ARBV such as DENV are important causes of fever, but attack rates can vary broadly from season to season or between relatively small distances. For example, 17.4% of 682 non-malaria febrile cases in a large health facility in Gabon were diagnosed as DENV, [18] and in Madagascar, DENV was the most common cause of AFI in one recent study. [55] Local epidemiology determines pre-test probability of a given diagnosis, which affects clinical decision making. Moreover, epidemiologic data permit priority setting and resource allocation by public health authorities. Detecting the true prevalence and incidence of ARBV infections can advocate for enhancing diagnostic capacity. Finally, the burden of ARBV infection represents a solvable global health problem. Vaccinology continues to advance, and it is likely that safe and effective vaccines could soon be available for ARBV including DENV, CHIKV and ZIKV. [56–60] There has also been reinvigoration of control efforts targeting mosquito vectors. [61–65] Effective implementation of these requires thorough knowledge of the local transmission ecology to design, monitor and maintain the benefit of novel interventions.

Limitations of our study include the relatively low sample amount, precluding more extensive serologic testing for other ARBV. For example, we did not test for IgM, which could have increased sensitivity for capturing acute ARBV infections. A longer longitudinal study would be necessary to capture cases of other ARBV, given the typical sporadic epidemic transmission patterns of these pathogens. Also, we were unable to collect follow up data on clinical outcomes of our DENV cases. Despite this constraint, we were able to generate a robust data set and provide new insight into ARBV transmission dynamics in SSA. DBS are an innovative tool that allows convenient sampling of large numbers of people in resource limiting setting. We and others have used DBS-based serology to study the epidemiology of arboviruses or other infectious diseases in both standard ELISA as well as multiplex platforms. [23,28,29,66–72] We have recently generated data on the stability of ARBV genomic RNA on DBS for RT-PCR diagnostic testing as well as metagenomic sequencing, [73] which will expand the possibilities for clinical studies and surveillance activities.

## Conclusion

In conclusion, this work highlights the need to consider DENV and other ARBV in the differential diagnosis of AFI in SSA. Our data indicate that western Cameroon is likely not hyperendemic for DENV, but sustained transmission of at least the DENV2 serotype in this region has likely been established for many years. Further study could also clarify if there is ongoing spill over from non-human mammalian hosts [48] or involvement of vector spp. other than *Aedes aegypti* and *albopictus* mosquitoes. [19,74] The methods used in this study provide an example of robust approaches to study ARBV. Optimal clinical management of febrile patients, along with efficiency and effectiveness of public health interventions such as vector control, would be strengthened were diagnostic capacity and surveillance systems improved and prioritized.

## Supporting information

S1 Table Symptoms reported among febrile patients. (DOCX)

S2 Table Reference sequences used for phylogenetic analysis. (XLSX)

S3 Table Site-specific testing data used in map creation. (DOCX)

S4 Table Full data set for molecular diagnostic testing by ZCD rRT-PCR assay. (XLSX)

S5 Table Sequencing metrics. (XLSX)

S1 Fig Amplification curves for positive samples from Cameroun in the DENV multiplex assay. **A-B)** Amplification curves for DENV-2 positive and negative samples in the DENV-2 (yellow) channel. **C**) Corresponding amplification curve from A shown in the DENV-4 (red) channel. Cross-reactions are readily distinguishable and serotype calls are made based on the pattern of fluorescence. Curves are color coded: pink, Cameroun samples; blue, DENV-2 control; red, DENV-4 control.
(TIF)

S2 Fig Maximum likelihood tree of 3,225 global DENV2 sequences. Sequences from this study (dark blue, arrow) cluster with other sequences from West Africa (medium blue) within the Cosmopolitan genotype (light blue). Other sequences are color-coded by genotype, and sequences belonging to the sylvatic lineage are in black.
(TIF)

S1 Text Supplementary Methods. (DOCX)
