# Supplementary material for: Arboviruses as an unappreciated cause of non-malarial acute febrile illness in the Dschang Health District of western Cameroon
Source: PLoS Negl Trop Dis. 2022 Oct 12;16(10):e0010790. doi: 10.1371/journal.pntd.0010790 (PMC9591055; doi:10.1371/journal.pntd.0010790)
Supplement: S1 Table — (DOCX) [file pntd.0010790.s001.docx]

**S1 Table**. Symptoms reported among febrile patients

| Symptom (n=311) | Number (%) |
| --- | --- |
| Headache | 127 (40.8) |
| General Fatigue | 122 (39.2) |
| Abdominal pain/diarrhoea | 154 (49.5) |
| Vomiting | 22 (7.1) |
| Cough | 22 (9.3) |
| Gastric Pain | 2 (0.6) |
| Other | 25 (8.0) |
