# Supplementary material for: Arboviruses as an unappreciated cause of non-malarial acute febrile illness in the Dschang Health District of western Cameroon
Source: PLoS Negl Trop Dis. 2022 Oct 12;16(10):e0010790. doi: 10.1371/journal.pntd.0010790 (PMC9591055; doi:10.1371/journal.pntd.0010790)
Supplement: S3 Table — (DOCX) [file pntd.0010790.s003.docx]

| S2 Table: Site-specific testing data used for map creation | | | | | | | |  |  |  |  |  |
| --- | --- | --- | --- | --- | --- | --- | --- | --- | --- | --- | --- | --- |
|  | **Dengue** | | | | | | |  | **Chikungunya** | | | |
|  | **PCR** | | | **Serology** | | | |  | **Serology** | | | |
| Location | Number tested | Positive | Negative | Number tested | Dengue only | Dengue + Chikungunya | Negative |  | Number tested | Chikungunya only | Chikungunya+ Dengue | Negative |
| BAF | 5 | 1 | 4 | 26 | 6 | 2 | 18 |  | 29 | 3 | 2 | 24 |
| BAL | 1 | 0 | 1 | 5 | 1 | 0 | 4 |  | 7 | 0 | 0 | 7 |
| DCE | 10 | 0 | 10 | 32 | 6 | 0 | 26 |  | 39 | 1 | 0 | 38 |
| FOK | 4 | 1 | 3 | 18 | 3 | 2 | 13 |  | 21 | 2 | 2 | 17 |
| FOR | 16 | 0 | 16 | 39 | 7 | 6 | 26 |  | 42 | 4 | 6 | 32 |
| FOT | 30 | 4 | 26 | 137 | 18 | 6 | 113 |  | 154 | 15 | 6 | 133 |
| KEL | 11 | 2 | 9 | 30 | 6 | 2 | 22 |  | 31 | 3 | 2 | 26 |
| SAT | 2 | 0 | 2 | 7 | 2 | 3 | 2 |  | 7 | 0 | 3 | 4 |
| TCH | 5 | 0 | 5 | 22 | 2 | 0 | 20 |  | 25 | 4 | 0 | 21 |
| TSI | 2 | 1 | 1 | 27 | 6 | 2 | 19 |  | 28 | 3 | 2 | 23 |
