# Supplementary figures and images for: Arboviruses as an unappreciated cause of non-malarial acute febrile illness in the Dschang Health District of western Cameroon
Source: PLoS Negl Trop Dis. 2022 Oct 12;16(10):e0010790. doi: 10.1371/journal.pntd.0010790 (PMC9591055; doi:10.1371/journal.pntd.0010790)

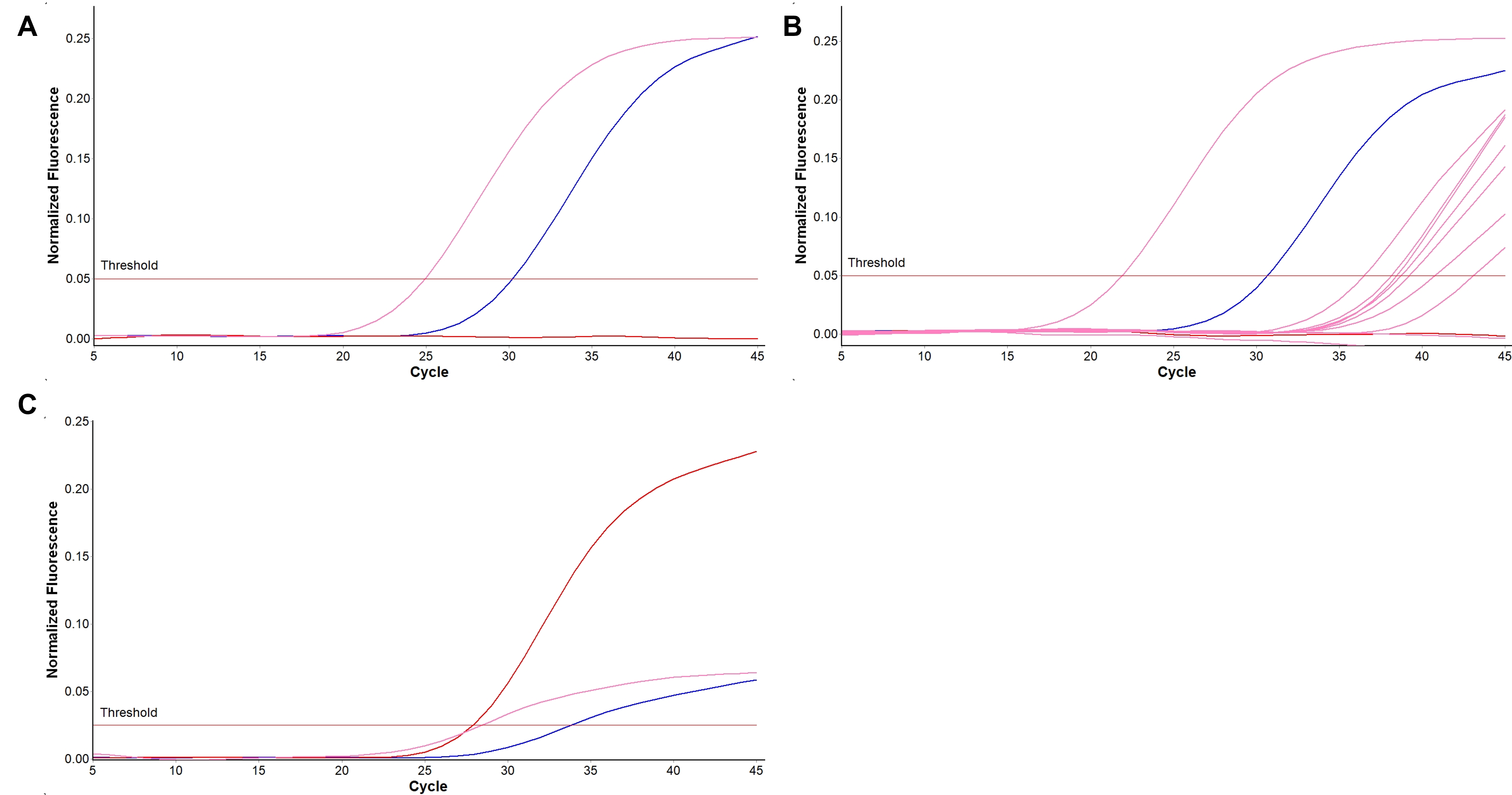

Supplement: S1 Fig — A-B) Amplification curves for DENV-2 positive and negative samples in the DENV-2 (yellow) channel. C) Corresponding amplification curve from A shown in the DENV-4 (red) channel. Cross-reactions are readily distinguishable and serotype calls are made based on the pattern of fluorescence. Curves are color coded: pink, Cameroun samples; blue, DENV-2 control; red, DENV-4 control. (TIF) [file pntd.0010790.s006.tif]

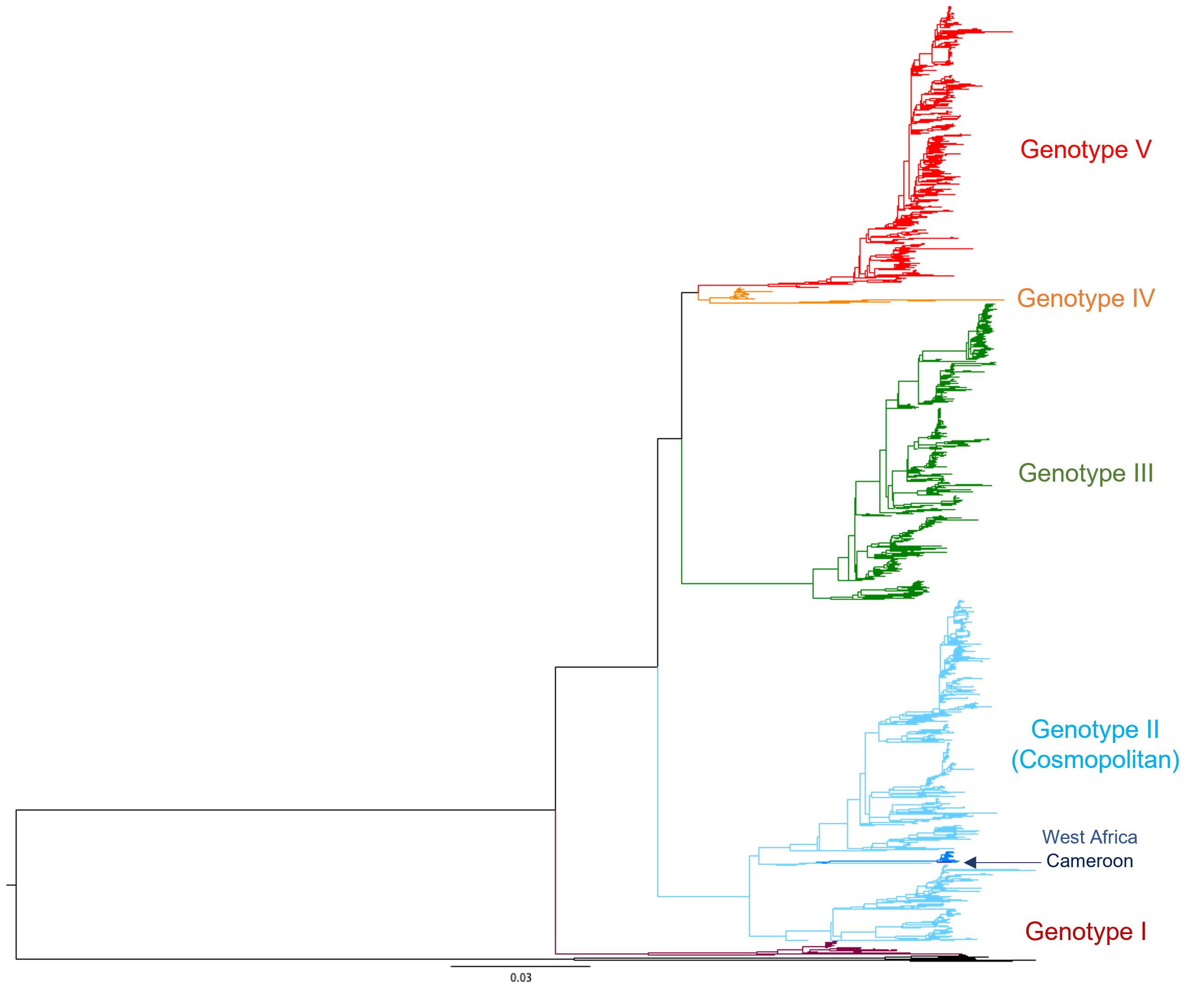

Supplement: S2 Fig — Sequences from this study (dark blue, arrow) cluster with other sequences from West Africa (medium blue) within the Cosmopolitan genotype (light blue). Other sequences are color-coded by genotype, and sequences belonging to the sylvatic lineage are in black. (TIF) [file pntd.0010790.s007.tif]
