# Supplementary material for: Arboviruses as an unappreciated cause of non-malarial acute febrile illness in the Dschang Health District of western Cameroon
Source: PLoS Negl Trop Dis. 2022 Oct 12;16(10):e0010790. doi: 10.1371/journal.pntd.0010790 (PMC9591055; doi:10.1371/journal.pntd.0010790)
Supplement: S1 Text — (DOCX) [file pntd.0010790.s008.docx]

SUPPLEMENTARY TEXT

Title: Arboviruses as an unappreciated cause of non-malarial acute febrile illness in the Dschang Health District of western Cameroon

**Supplementary Methods**

*Design*

This study was a prospective hospital based cross-sectional survey in the three main health facilities in the Dschang health district in the West region of Cameroon. Three clinical sites participated in this study: the Dschang district hospital, the St Vincent catholic hospital and the Batsinglah Catholic hospital. The Dschang health district with its 22 health areas has a surface area of about 1.060Km2. It is generally semi-urban, with typical relief features characterized by hills and slopes that render access to its interior geographically challenging. It is the largest health district in the western region of Cameroon. It is at an elevation of 1380m above sea level, with locations in our study area ranging 1380-1400m. Dschang has a tropical climate with rainy season between mid-March - mid October and a shorter dry season lasting about 5 months (November-March). The average temperature is 19.7 °C | 67.4 °F. Enrolment for this study was active from June 12, 2020 – September 8, 2020.

*Recruitment of study participants and biospecimens*

Eligible patients (n=560) were those undergoing screening for malaria as prescribed by the attending physician. Those who had fever (axillary temperature ≥37.5^O^C or history of fever in past 24 hours without signs and symptoms suggestive of severe malaria based on WHO criteria were not recruited due to the urgency in case management of these subjects. Eligible patients were approached for informed consent individually. Information on the study objectives and procedures were explained to the eligible subjects in French or English based on the preference of the subjects. A volunteer translator independent of the study team was available to subjects who could not read or write. Enrolled subjects were required to satisfy all inclusion criteria: 1) Male or female, 5-65 years of age 2) Presenting with fever (axillary temperature=37.5°C or above or self-reported in the last 24 hrs) 3) Willing and able to provide informed consent (consent was obtained from a parent if subject was less than age 21 and subjects older children also provided assent). Subjects were excluded if they had used anti-malarial medicine in the past 14 days. Informed consent was administered by trained study personnel in a separate room next to the hospital laboratory to ensure privacy is respected. This space is also used for post-test counselling of people tested for HIV-1 infection in the hospital.

All enrolled participants (n=431) were assigned a unique study code used to label all data and study material related to that patient. Blood specimens were obtained by finger prick with a sterile lancet and used for malaria smear microscopy and malaria rapid diagnostic test (RDT). Dried bloodspots (DBS) were also prepared. A questionnaire was then administered to collect socio-demographic data such as age, gender, neighbourhood/village as well as current symptoms, antimalarial treatment history, bednet use, location in the past three months. All specimens were first tested for malaria infection using a real-time PCR assay. Samples that were negative for malaria infection by this molecular assay were prioritized for rRT-PCR testing by a triplex assay detecting dengue (DENV), Zika (ZIKV), and chikungunya (CHIKV) viruses. We tested the first 96 subjects enrolled with available specimen and a negative malaria test result. Serology for DENV and CHIKV was performed on subjects with available sample, regardless of malaria testing status. Although participants were acutely ill at the time of enrollment in the study and sample collection, the IgG ELISA used is a better reflection of prior infection by *Aedes*-borne viruses (ABV) and not necessarily related to the etiology of the presenting illness.

The study was reviewed and approved by the IRB of the Cameroon Baptist Convention Health Board (FWA00002077), Protocol IRB2019-40.

*Site description, malaria study*

In total, 431 patients were enrolled in the study and distributed between the study hospitals: District Hospital Dschang (HDD, n=255 (59.3%)); Hopital Soeur servant du Christ de Batsengla (HSSCB, n=106 (24.6%)); Hopital Saint Vincent (HSV, n=70 (16.2%)). **Table 1** summarises key sociodemographic characteristics of the study population and association with ABV infection status. There were more females (58.1%) than males. The median age of the study population was 26 years, and children less than 10 years represented 10% of the total study population; the majority being adults aged 15-49 years old. Fifteen (5.9%) of 253 women were pregnant. Close to 53% of the study population had either secondary or tertiary education. With regards to occupation, only 9% had a job in the formal sector. The majority were students (jobless) [61% (56.9-66.4)]. While 86% reported travel out of the region (within Cameroon), most participants reported to have been in Dschang in the previous three months [77.9% (73.9-81.8)]. Most patients did not have a water source within 1km of their homes [82.3% (78.6-86.0)]. About ten percent of study participants reported keeping domestic animals at home. More than half of the study population [54.5% (50.1.-59.6)] reportedly owned bed nets. About the same proportion of all study participants slept under a bed net the previous night [55.2% (50.1.-59.6)] (Table 1). Symptomatology at presentation is reported in **Supplementary Table 1**. The most prevalent symptoms in addition to fever among the 311 patients endorsing any symptom were Abdominal pain or diarrhoea (49.5%), headache (40.8%), and general fatigue (39.2%). A minority of patients had cough, gastric pain, or other symptoms.

Clinical laboratory evaluations

*Microscopy*: A Giemsa stained thick and thin blood smear were made and examined by the study microscopist. Parasite density was calculated when the thick film was malaria parasite positive. The density estimation was based on a count of parasites per 200 WBCs (thick film) or per 5000 RBCs (thin film). Both asexual and sexual stages will be enumerated. A total of 200 oil immersion fields were examined on the thick film before a blood smear was considered negative.

*RDT*: Approximately 5μL of blood was transferred to a CareStart pLDH+HRP2-based malaria rapid diagnostic test cassette. Results were read 15 minutes after buffer was added based on manufacturer’s instructions. The participants who were parasite-positive by microscopy/RDT were referred for treatment based on local malaria case management guidelines. The DBS was temporarily stored at -20^o^C after drying overnight following spotting at the hospital. Two spots of about 75ul were made per participant. The DBS samples were shipped to the University of North Carolina for parasite speciation by PCR and to the University of Emory for serological screening, molecular diagnosis and whole genome sequencing of DENV positive cases from molecular diagnosis. Screening for exposure to other potential pathogens like Chikungunya, was done.

Laboratory assays

*rRT-PCR Testing:* Total nucleic acids were extracted from DBS on an EMAG instrument (bioMérieux, Durham, NC) by first placing 6mm punches into 400µL of Nuclisense lysis buffer and incubating overnight at room temperature on a tube rocker. Lysis buffer was then transferred to EMAG cartridges and extraction was performed using the standard protocol for serum/plasma according to manufacturer setting. Nucleic acids were eluted in 50µL of Buffer 3 and immediately tested by multiplex rRT-PCR for ZIKV, CHIKV, and DENV plus RNase P as the specimen control (the ZCD assay). Samples that generated a curve for DENV, regardless of Ct value up to cycle 45, were subsequently tested in a DENV multiplex assay to confirm detection and serotype the virus. The DENV multiplex assay contains primers and 4 molecular beacons that detect a region of the DENV 5’ UTR and *capsid*. This is the same region of the DENV genome targeted in the ZCD assay, though detection probes and chemistry are different. To be considered positive for DENV, a sample had to test positive for DENV RNA in both the ZCD and DENV multiplex assays. The ZCD and DENV multiplex assays were performed as described^1–3^ using 5µL of nucleic acid eluate. DENV serotype was determined from the pattern of signals generated in the DENV multiplex assay. In this assay, the channel for DENV4 yields detectable cross-reactions for samples with DENV2, as the sequences in the target region differ by a single base. Signals for DENV2 and DENV4 can be clearly distinguished based on the pattern of fluorescence (**S1 Fig**).

*DENV genome sequencing and phylogenetic analysis:* Seven samples underwent unbiased metagenomic sequencing followed by DENV-specific analysis. Extracted total nucleic acid was treated with heat-labile dsDNase (ArcticZymes, Tromso, Norway). cDNA was generated using random hexamer primers (Fisher/Invitrogen) and SUPERSCRIPT III RT (Fisher/Invitrogen) for first strand synthesis, and New England Biolabs reagents for second strand synthesis, without amplification. Sequencing libraries were fragmented, indexed, and amplified using the Nextera XT DNA Library Prep kit (Illumina) with dual unique indexes and 16 cycles of PCR. Libraries were quantified using the KAPA universal complete kit (Roche), pooled to equimolar concentration, and sequenced on a MiSeq with paired-end 150-bp reads (Illumina). As a negative control, water was included with each batch of samples starting from DNase. As a positive control, *in vitro* transcribed ERCC spike-ins (NIST) were added to each sample prior to cDNA synthesis.

Reads underwent metagenomic classification using Kraken and reference-based DENV genome assembly using reference MT261968, both implemented in viral-ngs version 2.0.21.3. For phylogenetic analysis, all complete DENV2 genomes were downloaded from NCBI on 7/3/22 (N=3,229), and a maximum-likelihood tree was constructed using a GTR+F+G4 model in IQ-TREE version 2.0.^4^ After Cameroon sequences were confirmed to cluster with other DENV2 sequences of the Cosmopolitan genotype from West Africa (Supplementary Figure S2), a subset of 29 West African DENV2 Cosmopolitan sequences were used for further analysis (Supplementary Table X: Reference sequences used for phylogenetic analysis). Sequence GQ398264 (Indonesia, 1976) was included as an outgroup. Sequences were aligned using MAFFT as implemented in Geneious (geneius.com) and trimmed to include the coding region only. A maximum-likelihood phylogenetic tree was constructed by model selection using a TIM2+F+G4 model with 1,000 bootstrap replicates in IQ-TREE version 2.0.^4^ Sequences were confirmed to have strong temporal structure using TempEst (correlation R-squared 99.8, slope 8.5 x 10^-4). Time-scaled Bayesian phylogeny was reconstructed from the full coding regions using BEAST^5^ with the following parameters: 100 million MCMC steps, GTR+gamma substitution model, 1+2+3 codon partitioning, relaxed clock with log-normal distribution, Bayesian Skyline. An MCC tree was generated using TreeAnnotator with a burn-in of 10%. Trees were visualized using FigTree (http://tree.bio.ed.ac.uk/software/figtree).

*Viruses and cells:* DENV WHO reference strains DENV1 West Pac 74, DENV2 S-16803, DENV3 CH53489, and DENV4 TVP-360 were initially obtained from Robert Putnak (Walter Reed Army Institute of Research, Silver Spring, Maryland, USA). CHIKV strain 181/clone25,^6^ which is a live attenuated vaccine strain compatible with BSL-2 work, was obtained from the World Reference Center for Emerging Viruses and Arboviruses (University of Texas Medical Branch, Galveston, Texas, USA). DENV stocks were prepared in C6/36 *Aedes albopictus* mosquito cells (ATCC no. CRL-1660) or Vero *Cercopithecus aethiops* monkey cells (ATCC no. CCL-81). C6/36 cells were grown at 32°C with 5% CO2 in minimum essential medium supple­mented with 10% fetal bovine serum (FBS), L-glutamine, nonessential amino acids, and HEPES (2-hydroxyethyl)-1-piperazineethanesulfonic acid) buffer. Vero cells were grown at 37°C with 5% CO2 in Dulbecco modified Eagle medium supplemented with 5% FBS and L-glutamine.

*Elution of dried blood spots for serologic analysis:* Plasma proteins were eluted from DBS as previously described.^7–9^ One 6-mm hole punch of each DBS was placed in a 1.5 mL Eppendorf tube with 300 μL of phosphate-buffered saline, and rotated for 2 hour at 37°C. This yielded an eluate that is equivalent to a 1:40 plasma dilution. Eppendorf tubes were centrifuged then eluate transferred to a new tube. Eluate was heat inactivated for 30 minutes in a 56°C water bath. The samples were centrifuged again to pellet proteinaceous debris and the supernatant was transferred to a new tube and stored at 4°C for up to 1 week or at -20°C until use.

*Antigen capture IgG ELISA:* Binding IgG to DENV or CHIKV was measured by antigen capture ELISA as previously described.^10,11^ Briefly, DENV antigen (an equal volume mixture of supernatant from each of the four DENV serotypes cultured in C6/36 cells) was captured by the anti-E protein mouse mAb 4G2.^12^ For CHIKV ELISA, plates were coated with the mAb CHK-48.^13^ Plates were blocked with 3% nonfat dry milk and incubated with DBS eluate at 37^o^C for 1 hour, and binding was detected with an alkaline phosphatase-conjugated goat anti-human IgG secondary Ab and *p*-nitrophenyl phosphate substrate. Absorbance at 405 nm (optical density, OD) was measured by spectrophotometry on a plate reader. ELISA data are reported as OD values that are the average of technical replicates. The average OD for technical replicates using DBS eluate obtained from flavivirus-naïve individuals (NHS) served as the negative control in ELISA assays. The cut off for positivity was calculated for each plate as the average OD of NHS = standard deviations + 0.1.^7–9^

*Neutralization Assays:* Neutralization titers were determined by 96-well micro focus reduction neutralization test (microFRNT) as previously described.^9,14,15^ Serial dilutions of DBS eluate were mixed with approximately 75-100 focus-forming units of virus in opti-MEM. The virus-antibody mixtures were incubated for 1 hour at 37°C and then transferred to a monolayer of Vero cells for infection for 2 hours at 37°C. Opti-MEM overlay media supplemented with 2% FBS and 5g (1%) Carboxymethylcellulose was then added, and cultures were incubated for 72 hours (DENV2) or 24 hours (CHIKV). Cells were fixed with 100 µL of 1:1 methanol:acetone for 30 minutes. 100 of blocking buffer (5% non-fat milk in pbs) 30 minutes at RT. 100 μL of 4G2^12^ (for DENV2) or CHK-48^13^ (for CHIKV) at 12.5 ng/μL were added to the plates and incubated for 2 hours at 37°C. Cells were washed with a microplate washer followed by the addition of 50 µl of 1:3000 horseradish peroxidase-conjugated goat anti-mouse secondary antibody for 1 hour at 37°C. Foci were visualized with 100 µL of True Blue and counted with a user-supervised automated counting program on 2x-magnified images of micro-wells obtained on a CTL ELISPOT reader. NHS controls were included on every plate to define 100% infection. To screen for DENV2 neutralizing antibodies, an abbreviated neutralization test was used (sdFRNT). DBS eluates were run at a single dilution (1:40). The sdFRNT was considered positive if the diluted serum neutralized ≥50% of input infectious viral particles (100% infection determined by virus only controls (VC)).

**References**

1. Waggoner, J. J. *et al.* Single-Reaction Multiplex Reverse Transcription PCR for Detection of Zika, Chikungunya, and Dengue Viruses. *Emerg. Infect. Dis.* **22**, 1295–1297 (2016).

2. JJ, W. *et al.* Single-reaction, multiplex, real-time rt-PCR for the detection, quantitation, and serotyping of dengue viruses. *PLoS Negl. Trop. Dis.* **7**, (2013).

3. JJ, W. *et al.* Comparison of the FDA-approved CDC DENV-1-4 real-time reverse transcription-PCR with a laboratory-developed assay for dengue virus detection and serotyping. *J. Clin. Microbiol.* **51**, 3418–3420 (2013).

4. Nguyen, L. T., Schmidt, H. A., Von Haeseler, A. & Minh, B. Q. IQ-TREE: a fast and effective stochastic algorithm for estimating maximum-likelihood phylogenies. *Mol. Biol. Evol.* **32**, 268–274 (2015).

5. Rambaut, A. Estimating the rate of molecular evolution: incorporating non-contemporaneous sequences into maximum likelihood phylogenies. *Bioinformatics* **16**, 395–399 (2000).

6. NH, L. *et al.* Development of an attenuated strain of chikungunya virus for use in vaccine production. *Vaccine* **4**, 157–162 (1986).

7. Tissera, H. *et al.* Burden of dengue infection and disease in a pediatric cohort in urban Sri Lanka. *Am. J. Trop. Med. Hyg.* **91**, 132–137 (2014).

8. Corbett, K. S. *et al.* Preexisting neutralizing antibody responses distinguish clinically inapparent and apparent dengue virus infections in a Sri Lankan pediatric cohort. *J. Infect. Dis.* **211**, 590–599 (2015).

9. Willcox, A. C. *et al.* Seroepidemiology of Dengue, Zika, and Yellow Fever Viruses among Children in the Democratic Republic of the Congo. *Am. J. Trop. Med. Hyg* **99**, 756–763 (2018).

10. de Alwis, R. *et al.* Identification of human neutralizing antibodies that bind to complex epitopes on dengue virions. *Proc. Natl. Acad. Sci. U. S. A.* **109**, 7439–7444 (2012).

11. Collins, M. H. *et al.* Human antibody response to Zika targets type-specific quaternary structure epitopes. *JCI Insight* **4**, (2019).

12. Henchal, E. A., Gentry, M. K., McCown, J. M. & Brandt, W. E. Dengue virus-specific and flavivirus group determinants identified with monoclonal antibodies by indirect immunofluorescence. *Am. J. Trop. Med. Hyg.* **31**, 830–836 (1982).

13. Fox, J. M. *et al.* Broadly Neutralizing Alphavirus Antibodies Bind an Epitope on E2 and Inhibit Entry and Egress. *Cell* **163**, 1095–1107 (2015).

14. Collins, M. H. *et al.* Lack of Durable Cross-Neutralizing Antibodies Against Zika Virus from Dengue Virus Infection. *Emerg. Infect. Dis.* **23**, 773–781 (2017).

15. Collins, M. H. *et al.* Serologic surveillance of maternal Zika infection in a prospective cohort in Leon, Nicaragua during the peak of the Zika epidemic. *PLoS One* **15**, (2020).
